# Three-year follow-up of a phase II study of radium-223 dichloride in Japanese patients with symptomatic castration-resistant prostate cancer and bone metastases

**DOI:** 10.1007/s10147-018-01389-4

**Published:** 2019-03-14

**Authors:** Hirotsugu Uemura, Hiroji Uemura, Satsohi Nagamori, Yoshiaki Wakumoto, Go Kimura, Hiroaki Kikukawa, Akira Yokomizo, Atsushi Mizokami, Takeo Kosaka, Naoya Masumori, Yoshihide Kawasaki, Junji Yonese, Yasutomo Nasu, Satoshi Fukasawa, Takayuki Sugiyama, Seigo Kinuya, Makoto Hosono, Iku Yamaguchi, Takashi Akagawa, Nobuaki Matsubara

**Affiliations:** 1 Department of Urology, Kindai University Faculty of Medicine, 377-2, Ohno-Higashi, Osaka-Sayama, Osaka 589-8511 Japan; 2 Department of Urology and Renal Transplantation, Yokohama City University Medical Center, 4-57, Urafune-cho, Minami-ku, Yokohama, Japan; 3 Department of Urology, National Hospital Organization Hokkaido Cancer Center, 2-3-54 Kikusui 4 Jo, Shiroishi-ku, Sapporo, Japan; 4 Department of Urology, Juntendo University, 2-2-1 Hongo Bunkyo-ku, Tokyo, Japan; 5 Department of Urology, Nippon Medical School, 1-1-5, Sendagi, Bunkyo-ku, Tokyo, Japan; 6 Department of Urology, National Hospital Organization Kumamoto Medical Center, 1-5 Ninomaru, Chuo-ku, Kumamoto, Japan; 7 Department of Urology, Graduate School of Medical Sciences, Kyushu University, 3-1-1 Maidashi, Higashi-ku, Fukuoka, Japan; 8 Department of Integrative Cancer Therapy and Urology, Kanazawa University Graduate School of Medical Science, 13-1 Takaramachi, Kanazawa, Ishikawa Japan; 9 Department of Urology, Keio University School of Medicine, 35 Shinanomachi, Shinjuku-ku, Tokyo, Japan; 10 Department of Urology, Sapporo Medical University School of Medicine, South 1, West 16, Chuo-ku, Sapporo, Japan; 11 Department of Urology, Tohoku University Hospital, 1-1, Seiryo-machi, Aoba-ku, Sendai, Japan; 12 Department of Urology, Cancer Institute Hospital of Japanese Foundation for Cancer Research, 3-8-31, Ariake, Koto-ku, Tokyo, Japan; 13 Department of Urology, Dentistry and Pharmaceutical Sciences, Okayama University Graduate School of Medicine, 2-5-1, Shikata, Okayama Japan; 14 Prostate Center, Division of Urology, Chiba Cancer Center, 666-2, Nitona-cho, Chuo-ku, Chiba, Japan; 15 Department of Urology, Hamamatsu University School of Medicine, 1-20-1, Handayama, Higashi-ku, Hamamatsu, Japan; 16 The Japanese Society of Nuclear Medicine, 2-28-45, Honkomagome, Bunkyo-ku, Tokyo, Japan; 17 Clinical Statistics, Bayer Yakuhin, Ltd, 2-4-9, Umeda, Kita-ku, Osaka, Japan; 18 Oncology Clinical Development, Bayer Yakuhin, Ltd, 2-4-9, Umeda, Kita-ku, Osaka, Japan; 19 Division of Breast and Medical Oncology, National Cancer Center Hospital East, 6-5-1 Kashiwanoha, Kashiwa, Chiba Japan

**Keywords:** Bone metastases, Metastatic castration-resistant prostate cancer, Overall survival, Radium-223 dichloride, Safety

## Abstract

**Background:**

Radium-223 is a first-in-class targeted alpha therapy to prolong overall survival (OS) in castration-resistant prostate cancer with bone metastases (mCRPC). The aim of the present analysis was to assess the long-term safety with radium-223 in Japanese patients with mCRPC.

**Methods:**

Patients with symptomatic mCRPC, ≥ 2 bone metastases and no known visceral metastases received up to 6 injections of radium-223 (55 kBq/kg), one every 4 weeks. Adverse events (AEs) considered to be related to radium-223 were reported until 3 years after the first injection. Pre-specified conditions, such as acute myelogenous leukemia, myelodysplastic syndrome, aplastic anemia, primary bone cancer, or other primary malignancies, were reported regardless of causality.

**Results:**

Of the 49 patients enrolled in the study, 44 (89.8%) entered the survival follow-up period and 33 (67.3%) died. Throughout the entire study, there were no reports of second primary malignancy or other pre-specified conditions. Eight patients (16.3%) experienced post-treatment drug-related AEs, which were all hematological (anemia and decreased lymphocyte, platelet, and white blood cell counts). No serious post-treatment drug-related AEs were reported. Updated median OS was 19.3 months (95% CI: 14.2, 28.5).

**Conclusions:**

In Japanese patients with symptomatic mCRPC and bone metastases, radium-223 had a favorable long-term safety profile with no second primary malignancies reported. Taken together with median OS, which was comparable to that in the pivotal phase III ALSYMPCA study, these results support continued benefit from radium-223 in Japanese patients with mCRPC.

**Electronic supplementary material:**

The online version of this article (10.1007/s10147-018-01389-4) contains supplementary material, which is available to authorized users.

## Introduction

Bone is one of the most common metastatic sites among patients with prostate cancer [1]. In patients with metastatic castration-resistant prostate cancer (mCRPC), bone metastases are associated with skeletal-related events (SREs), including fractures, that reduce quality of life [2] and shorten overall survival (OS) [3]. Bisphosphonates (e.g., zoledronic acid) and denosumab have been shown to decrease the risk of SREs and strontium-89 has been shown to alleviate pain in patients with mCRPC and bone metastases [4–6]. However, none of these treatments have provided significant improvements in OS [7].

Radium-223 dichloride (radium-223) is a first-in-class targeted alpha therapy that has been developed for the treatment of skeletal metastases [7, 8]. Due to its chemical similarity to calcium, radium-223 is absorbed into the bone, primarily in the areas with high metabolic activity [8–10]. The randomized phase III ALSYMPCA study, conducted in 921 patients with mCRPC and symptomatic bone metastases, demonstrated significantly prolonged OS with radium-223 vs placebo when added to best standard of care (BSoC; *p* = 0.002) [11]. In ALSYMPCA, radium-223 was also associated with a significantly prolonged time to symptomatic skeletal events (SSEs) and reduced alkaline phosphatase (ALP) levels vs placebo (*p* < 0.001 for both), and was well tolerated [11]. In addition, no second primary treatment-related malignancies were reported when patients were followed for up to 3 years [12].

In a phase II study of Japanese patients with symptomatic mCRPC and bone metastases (*n* = 49), intravenous radium-223 was associated with a mean change in total ALP from baseline to 12 weeks (primary endpoint) of − 19.3% (95% confidence interval [CI]: − 28.0, − 10.7) [13]. The ALP change was considered to be consistent with that in ALSYMPCA. After a median of 8.5 months of follow-up, the 1-year OS and SSE-free rates were 78% and 89%, respectively. Grade 3 or 4 treatment-emergent adverse events (TEAEs) with an incidence of ≥ 10% were decreased lymphocyte count (14%), anemia (14%), anorexia (10%), and bone pain (10%) [13].

The aim of the present analysis was to assess the long-term safety, as well as updated OS, associated with radium-223 in Japanese patients with symptomatic mCRPC and bone metastases enrolled in this phase II study using follow-up data of 3 years since the first administration.

## Patients and methods

### Study design

The study protocol was approved by each study center’s independent ethics committee or institutional review board, and the study was conducted in accordance with the ethical principles of the Declaration of Helsinki and the International Conference on Harmonization guideline E6: Good Clinical Practice. Written informed consent was obtained from all patients.

The design of this multicenter, single-arm, open-label, phase II study (NCT01929655) has been previously described [13]. Briefly, eligibility criteria were similar to those of ALSYMPCA [11] and patients with progressive, symptomatic CRPC, ≥ 2 bone metastases, and no known visceral metastases treated in Japan were enrolled. Patients were required to have a history of, refused, or be ineligible for docetaxel. Concomitant BSoC was permitted, including external beam radiotherapy (EBRT), corticosteroids, first-generation anti-androgens such as flutamide, estrogens, ketoconazole, bisphosphonates, and denosumab. During the treatment period, cytotoxic chemotherapy, other systemic radioisotopes, hemibody irradiation, and other investigational drugs, such as abiraterone and enzalutamide, were not permitted.

### Study treatment and follow-up

All enrolled patients received intravenous radium-223 at a dose of 55 kBq/kg on day 1 of each 4-week cycle for a maximum of six cycles.

The study consisted of a treatment period, defined as the time from the first injection to end of treatment (EOT; visit that occurred 4 weeks after the 6th injection or within 2 weeks after discontinuation), an active follow-up period (from EOT to 12 weeks thereafter), and a survival follow-up period (from 12 weeks after EOT to 3 years after the first injection). Efficacy data, as well as SSEs, were assessed at visits conducted on day 1 of each treatment cycle, at EOT, and once every 4 weeks during the active follow-up period. Concomitant or subsequent therapy started within 30 days after the last injection was recorded. During the survival follow-up period, patients were contacted every 6 months, either over the telephone or by visits, and survival was assessed.

TEAEs (AEs that occurred after the first injection of radium-223, but within 30 days after the last injection) were reported regardless of causality. Investigators were required to report post-treatment AEs, which occurred more than 30 days after the last injection, only if they were considered drug-related (post-treatment drug-related AE). All instances of acute myeloid leukemia (AML), myelodysplastic syndrome, aplastic anemia, and bone cancer, as well as any other second primary malignancies, were considered serious AEs (SAEs) and were reported regardless of the time of occurrence or causality.

### Statistical analyses

Statistical analyses were performed using the software package SAS release 9.2. The Kaplan–Meier method was used to estimate median OS, while median survival follow-up time was estimated by the reverse Kaplan–Meier method [14]. Descriptive subgroup analyses of OS based on several baseline parameters were conducted (post hoc exploratory analyses). Median OS and 95% CIs were estimated using the Kaplan–Meier method according to the following baseline clinical characteristics and laboratory variables: age; European Cooperative Oncology Group performance status (ECOG PS); extent of disease (EOD); Gleason score; time since initial prostate cancer diagnosis; time since bone metastasis diagnosis; time since first progression of prostate cancer; time since first diagnosis of bone metastases; prior use of docetaxel; prior palliative radiotherapy; concomitant use of bone-modifying agents; lactate dehydrogenase (LDH); total ALP; bone alkaline phosphatase (BAP); prostate-specific antigen (PSA); procollagen 1 amino-terminal propeptide (P1NP); type 1 collagen degradation product (1CTP); C-terminal cross-linked telopeptide of type 1 collagen (CTX-1); albumin; hemoglobin; neutrophils; and platelets. To minimize the difference in the number of patients between the two groups, a dichotomization cutoff was set at the median or the nearest value to it. Hazard ratios were estimated based on the Cox proportional hazards model.

## Results

### Patient disposition and treatment

**Fig. 1:**
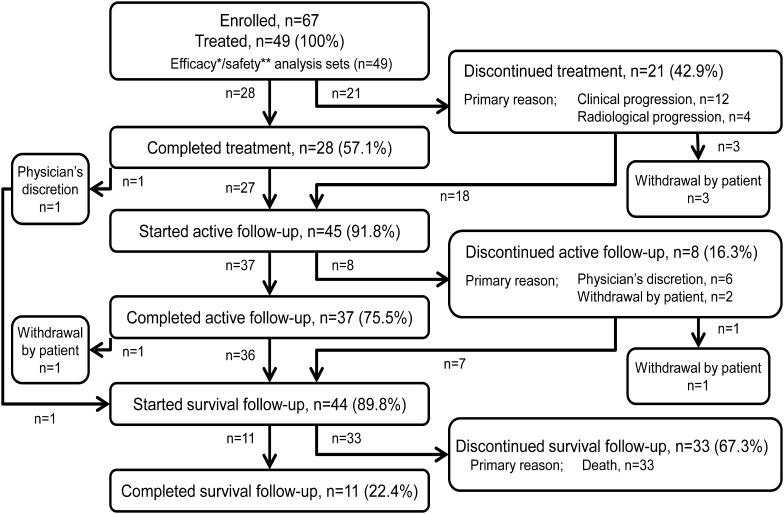
Patient flow diagram

Of the 49 patients with mCRPC who were administered radium-223, 45 (91.8%) entered the active follow-up period, while 4 patients (8.2%) did not (Fig. 1). Of these 45 patients, 8 (16.3%) discontinued and 37 (75.5%) completed the active follow-up period. Overall, 44 patients (89.8%) entered the survival follow-up period, which was continued up to 3 years after the first administration of radium-223. By the end of the survival follow-up period, 33 patients (67.3%) had died, while 11 (22.4%) remained alive.

A summary of all anticancer drugs that were started within 30 days after the last radium-223 dose are listed in Supplementary Table 1.

### Safety

Throughout the study, there were no reports of AML, myelodysplastic syndrome, aplastic anemia, bone cancers, or any other second primary malignancies.

Of the 49 patients in the safety analysis set (including three patients who did not enter either the active or survival follow-up period), 8 patients (16.3%) experienced post-treatment drug-related AE; all of these events were hematologic toxicities (Table 1). The incidence of Grade 1, Grade 2, and Grade 3 post-treatment drug-related AEs was 2.0%, 8.2%, and 6.1%, respectively. None of the post-treatment drug-related AEs were serious. All patients who experienced a post-treatment drug-related AE had experienced the same event during the treatment period. However, in three patients, the severity of drug-related AEs changed from Grade 1–2 during the treatment period to Grade 3 thereafter (decreased lymphocyte count, *n* = 1; decreased platelet count, *n* = 2), while 17 and 10 patients experienced Grade 1–2 and Grade 3–4 drug-related AEs in treatment period. Of these three patients, one experienced clinical disease progression and discontinued radium-223 at the occurrence of the AE (decreased platelet count). The use of post-radium chemotherapy before worsening of the AE was not reported for any of these patients. Due to the fact that TEAEs and post-treatment drug-related AEs were collected differently, the data on the incidence of drug-related TEAEs are presented for reference only and include only the AEs which were also reported as post-treatment drug-related AEs. Voluntarily reported non-related AEs are summarized in Supplementary Table 2.

**Table 1:** Drug-related adverse events after radium-223 dichloride treatment in the safety analysis set (*n* = 49)

Drug-related AE, *n* (%)	Post-treatment drug-related AE^a, b^		Treatment-emergent drug-related AE^c^ (for reference only)		
	All grades	Grade 3	All grades	Grade 3	Grade 4
Any	8 (16.3)	3 (6.1)	27 (55.1)	9 (18.4)	1 (2.0)
Lymphocyte count decreased	4 (8.2)	2 (4.1)	12 (24.5)	5 (10.2)	1 (2.0)
Platelet count decreased	3 (6.1)	2 (4.1)	6 (12.2)	1 (2.0)	0
Anemia	3 (6.1)	1 (2.0)	15 (30.6)	6 (12.2)	0
White blood cell decreased	1 (2.0)	0	4 (8.2)	0	0

^a^Drug-related AEs which occurred > 30 days after the last injection to 3 years after the first injection

^b^Safety analysis set included the 3 patients who did not enter either of active- and survival follow-up

^c^From the first injection of radium-223 to 30 days after the last injection. Only the events that also reported as post-treatment drug-related AE are shown for the reference purpose

During the entire study period, fractures were reported in only three patients (Supplementary Table 3). Two patients experienced fractures during the treatment period (6 and 47 days after the first injection of radium-223), and one during the active follow-up period (131 days after the first injection, 47 days after the last injection). None of the fractures were considered serious or related to the study drug. All fractures were classified as Grade 2 AEs. None of the patients who had fractures had used bone-modifying agents, either before or concomitantly with study drugs.

### Overall survival

**Fig. 2:**
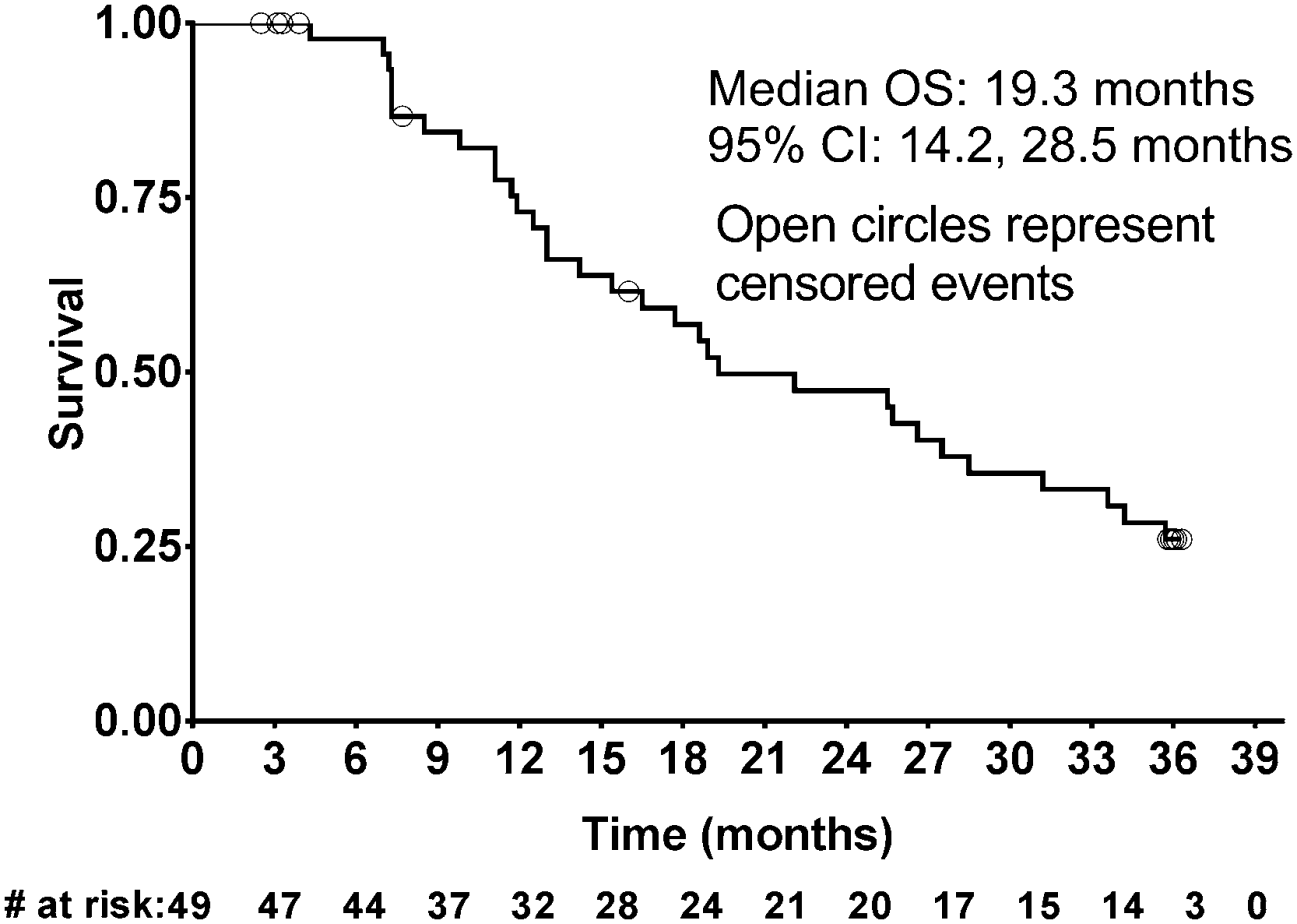
Kaplan–Meier curve for overall survival (OS). CI, confidence interval

After a median follow-up time of 35.9 months, 32 death events had occurred. In addition, 1 patient with an unknown date of death was censored. The median OS was 19.3 months (95% CI: 14.2, 28.5; Fig. 2), while the 6-month and 1-year OS rates were 98% (95% CI: 85, 100) and 73% (95% CI: 57, 84), respectively.

**Fig. 3:**
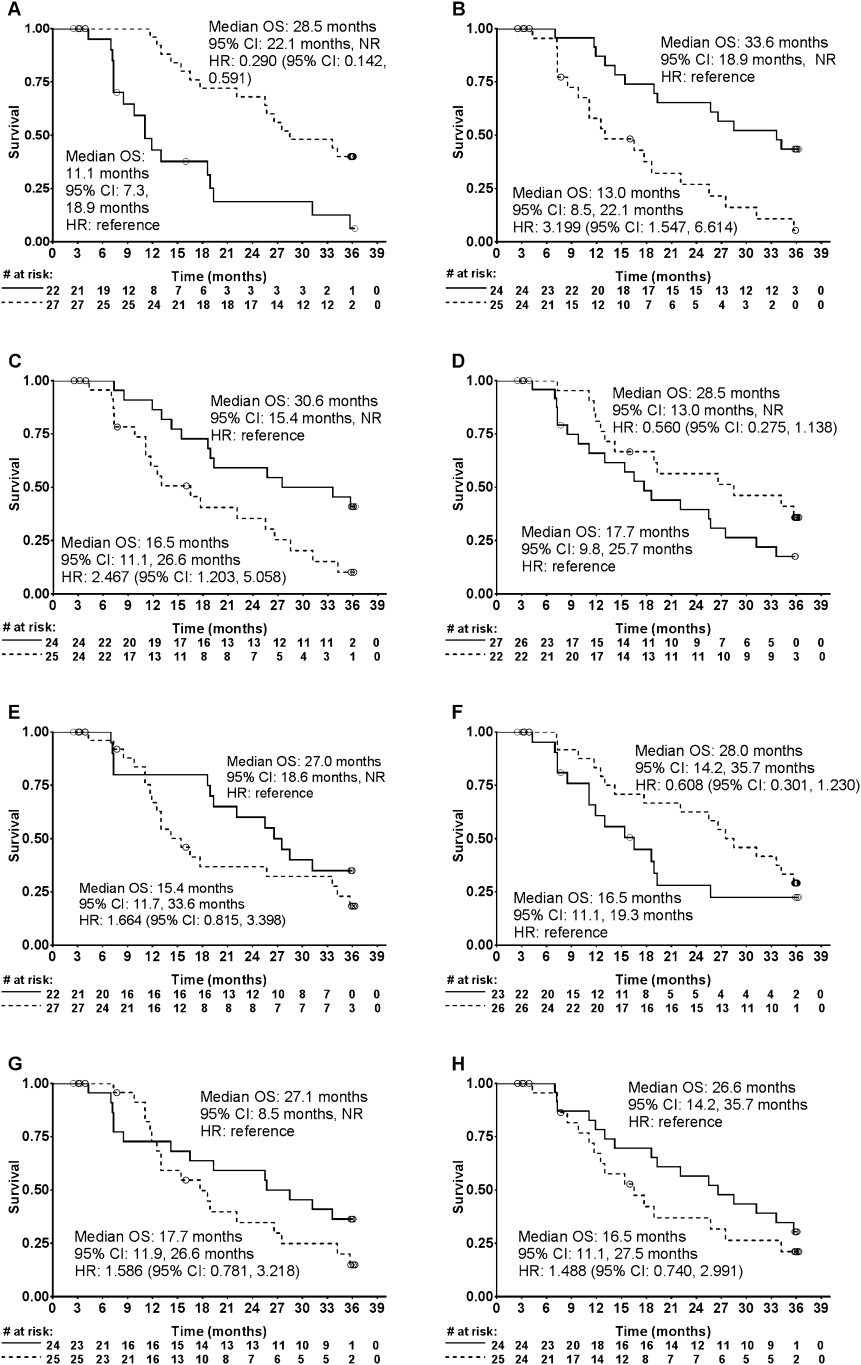
Kaplan–Meier curve for overall survival (OS) by baseline **a** albumin levels (< median [3.9 g/dL; solid line] vs ≥ median [dashed line]), **b** neutrophils (< median [4550 µL; solid line] vs ≥ median [dashed line]), **c** lactate dehydrogenase (< median [212 U/L; solid line] vs ≥ median [dashed line]), **d** prior use of docetaxel (yes [solid line] vs no [dashed line]), **e** extent of disease (1–2 [solid line] vs 3–4 [dashed line]), **f** hemoglobin (< median [11.8 g/dL; solid line] vs ≥ median [dashed line]), **g** total alkaline phosphatase (< median [335 U/L; solid line] vs ≥ median [dashed line]), **h** prostate specific antigen (< median [101 U/L; solid line] vs ≥ median [dashed line]). Only subgroups with hazard ratio or its reciprocal < 0.7 are shown. *CI* confidence interval, *HR* hazard ratio, *NR* not reached

Subgroup analyses of OS were performed based on patient baseline characteristics. Median values for parameters were arbitrarily chosen as the cut off for the subgroups. Kaplan–Meier curves were depicted for those with hazard ratio or its reciprocal of less than 0.7 (Fig. 3); in these figures, a trend for longer median OS was seen with higher baseline albumin, lower neutrophil counts, lower LDH levels, without prior history of treatment with docetaxel, EOD scores of 1–2 vs 3–4, higher hemoglobin, lower total ALP and PSA (Table 2).

**Table 2:** Subgroup analyses of overall survival with radium-223 dichloride by baseline patient characteristics and laboratory parameters in Japanese patients with metastatic castration-resistant prostate cancer and symptomatic bone metastases

Factor	Subgroup	*n*	Median OS, months (95% CI)	Hazard ratio^a^	OS ratio at 12 months (95% CI)
Age (median 74 years)	< 75 years	25	16.5 (11.1, 33.6)	Reference	0.64 (0.41, 0.80)
	≥ 75 years	24	22.5 (13, N/E)	0.761 (0.379, 1.526)	0.82 (0.59, 0.93)
ECOG PS	0	36	22.1 (14.2, 28.5)	Reference	0.75 (0.57, 0.87)
	1–2	13	19.1 (7.2, N/E)	0.871 (0.390, 1.943)	0.67 (0.34, 0.86)
EOD	1–2	22	27 (18.6, N/E)	Reference	0.8 (0.55, 0.92)
	3–4	27	15.4 (11.7, 33.6)	1.664 (0.815, 3.398)	0.67 (0.45, 0.82)
Gleason score	≤8	23	22.1 (12.5, N/E)	Reference	0.74 (0.51, 0.87)
	> 8	26	16.5 (11.7, 31.2)	1.423 (0.706, 2.867)	0.72 (0.47, 0.86)
Time since initial diagnosis of PCa (median 3.9 years)	< Median	25	19.3 (11.7, 28.5)	Reference	0.68 (0.45, 0.83)
	≥Median	24	25.7 (13, 34.2)	1.034 (0.516, 2.073)	0.78 (0.55, 0.90)
Time since initial diagnosis of bone metastases (median 2.3 years)	< Median	25	18.9 (13, 33.6)	Reference	0.75 (0.53, 0.88)
	≥Median	24	25.5 (11.9, N/E)	0.854 (0.424, 1.719)	0.71 (0.46, 0.86)
Time since first progression of PCa (median 2.4 years)	< Median	24	19.3 (11.7, 28.5)	Reference	0.68 (0.45, 0.83)
	≥Median	25	25.7 (13, 35.7)	0.893 (0.446, 1.790)	0.78 (0.55, 0.90)
ALP (median 335 U/L)	< Median	24	27.1 (8.5; N/E)	Reference	0.73 (0.49, 0.87)
	≥Median	25	17.7 (11.9, 26.6)	1.586 (0.781, 3.218)	0.73 (0.49, 0.87)
PSA (median 101 μg/L)	< Median	24	26.6 (14.2, 35.7)	Reference	0.78 (0.55, 0.90)
	≥Median	25	16.5 (11.1, 27.5)	1.488 (0.740, 2.991)	0.67 (0.43, 0.83)
LDH (median 212 U/L)	< Median	24	30.6 (15.4, N/E)	Reference	0.86 (0.63, 0.95)
	≥Median	25	16.5 (11.1, 26.6)	2.467 (1.203, 5.058)	0.6 (0.37, 0.77)
BAP (median 24.5 μg/L)	< Median	24	22.4 (11.9, 33.6)	Reference	0.73 (0.49, 0.87)
	≥Median	25	18.9 (11.7, 34.2)	1.038 (0.518, 2.078)	0.73 (0.50, 0.87)
P1NP (median 49.7 μg/L)	< Median	24	23.8 (8.5, 33.6)	Reference	0.68 (0.45, 0.83)
	≥Median	25	17.7 (12.5, 34.2)	0.887 (0.442, 1.781)	0.77 (0.54, 0.90)
1CTP (median 5.9 μg/L)	< Median	23	25.6 (13, 33.6)	Reference	0.77 (0.54, 0.90)
	≥median	26	16.5 (11.7, N/E)	1.031 (0.513, 2.073)	0.69 (0.45, 0.84)
CTX-I (median 0.11 μg/L)	< Median	23	22.1 (13; 28.5)	Reference	0.76 (0.52, 0.89)
	≥Median	26	18.6 (11.9, N/E)	0.822 (0.409, 1.651)	0.7 (0.47, 0.84)
Hemoglobin (median 11.8 g/dL)	< Median	23	16.5 (11.1, 19.3)	Reference	0.61 (0.36, 0.78)
	≥Median	26	28 (14.2, 35.7)	0.608 (0.301, 1.230)	0.83 (0.61, 0.93)
Neutrophil count (median 4550/μL)	< Median	24	33.6 (18.9, N/E)	Reference	0.87 (0.65, 0.96)
	≥Median	25	13 (8.5, 22.1)	3.199 (1.547, 6.614)	0.58 (0.35, 0.76)
Platelet count (median 223,000/μL)	< Median	24	26.6 (15.4, 35.7)	Reference	0.74 (0.51, 0.87)
	≥Median	25	16.5 (11.9, 31.2)	1.276 (0.636, 2.560)	0.72 (0.48, 0.86)
Albumin (median 3.9 g/dL)	< Median	22	11.1 (7.3, 18.9)	Reference	0.43 (0.21, 0.63)
	≥Median	27	28.5 (22.1, N/E)	0.290 (0.142, 0.591)	0.96 (0.75, 0.99)
Prior use of docetaxel	Yes	27	17.7 (9.8, 25.7)	Reference	0.66 (0.43, 0.81)
	No	22	28.5 (13, N/E)	0.560 (0.275, 1.138)	0.81 (0.57, 0.92)
Prior radiotherapy (palliative)	Yes	14	23.8 (11.1, N/E)	Reference	0.79 (0.47, 0.93)
	No	35	18.9 (13, 33.6)	1.093 (0.517, 2.311)	0.7 (0.51, 0.83)
Concomitant use of bone-modifying agents	Yes	31	25.5 (16.5, 33.6)	Reference	0.85 (0.65, 0.94)
	No	18	14 (8.5, 34.2)	1.222 (0.602, 2.483)	0.56 (0.31, 0.75)

*1CTP* type 1 collagen degradation product, *ALP* alkaline phosphatase, *BAP* bone alkaline phosphatase, *CI* confidence interval, *CTX-I* C-terminal cross-linked telopeptide of type 1 collagen, *ECOG PS* European Cooperative Oncology Group performance status, *EOD* extent of disease, *LDH* lactate dehydrogenase, *N/E* not estimated due to censored data, *OS* overall survival, *P1NP* procollagen 1 amino-terminal propeptide, *PCa* prostate cancer, *PSA* prostate-specific antigen

^a^Estimated based on the Cox proportional hazards model; hazard ratio =(hazard with the subgroup of interest)/(hazard with reference subgroup)

## Discussion

Radium-223 is the first-in-class targeted alpha therapy to show prolonged OS in patients with mCRPC and bone metastases, as demonstrated in the pivotal ALSYMPCA study [11]. In ALSYMPCA, median OS was 14.9 months with radium-223 vs 11.3 months with placebo. Radium-223 was well tolerated, with a low incidence of myelosuppression, and 3-year follow-up added no new safety concerns. In addition, there were no instances of second primary malignancies due to radium-223 treatment [11, 12].

In the present report, the incidence of post-treatment drug-related AEs after radium-223 therapy was generally low and there were no new safety concerns. All post-treatment drug-related AEs were hematologic in nature. The incidence of hematologic AEs during the follow-up period was marginally higher in the present study than in ALSYMPCA (decreased lymphocyte count: 8% vs 0%; decreased platelet count: 6% vs 1%; anemia: 6% vs 3%; respectively), Grade 3 AEs were relatively infrequent (2–4%) in the present analysis. In addition, all patients who reported these events had already experienced the same hematologic AEs during the treatment period. This indicates that late-onset new hematologic AEs with radium-223 are rare.

Because radiotherapy, for example EBRT, increases the risk of secondary malignancies [15], radium-223 may potentially also increase this risk. However, in the present study, no second primary malignancies were observed. This is consistent with ALSYMPCA, in which the incidence of post-treatment primary malignancies (regardless of causality) did not increase in the radium-223 treatment arm compared with the placebo arm. However, because of the relatively short observational period of the present study and ALSYMPCA, partly due to the limited survival of patients, the possibility of an increased risk of malignancy with radium-223 cannot be completely ruled out. Furthermore, as one patient in the radium-223 arm of ALSYMPCA experienced aplastic anemia during the follow-up [12], a potentially increased risk of bone marrow disease and hematologic malignancies cannot be dismissed.

In another phase 3 study (ERA 223), in which radium-223 or placebo was initiated at the same time as abiraterone acetate plus prednisone/prednisolone in asymptomatic or mildly symptomatic patients with mCRPC and bone metastases [16], preliminary data indicated a higher incidence of fractures and death in patients who received a combination of radium-223 plus abiraterone and prednisone/prednisolone [17]. In the present study, in which new hormonal agents were not used concomitantly with radium-223, fractures were reported in only three patients during the study period. Reported fractures were all Grade 2, non-serious, and non-treatment-related. None of the patients who had fractures had used bone-modifying agents, either before or concomitantly with study drugs. This may imply the importance of using bone-modifying agents during mCRPC treatment, including radium-223, which is also recommended in many guidelines for prostate cancer including those from the Japanese Urological Association [18–20]. It should be noted that the data on fractures may not have been fully captured after the treatment period because the definition of SSEs, which were collected until the active follow-up period, included only pathological fractures and because only drug-related AEs were collected during the active and survival follow-up periods.

In the primary analysis of this phase II study of Japanese patients with symptomatic mCRPC and bone metastases, decreased ALP levels at 12 weeks (primary endpoint) were observed following radium-223 therapy [13], consistent with the pivotal ALSYMPCA study [21]. While it shows the presence of the pharmacodynamic action of radium-223 in Japanese patients, the duration of the primary analysis was insufficient to evaluate the efficacy outcomes in this patient population. In the present analysis, a median OS of 19.3 months was obtained after a longer median follow-up of 35.9 months. This is numerically longer than the OS observed in ALSYMPCA (14.9 months) [11]. Although a simple comparison between unmatched cohorts is not appropriate, particularly as a difference in background or post-radium-223 therapies may have affected OS, these results suggest that an OS benefit with radium-223 can be expected in Japanese patients.

In the exploratory subgroup analyses, a trend for a longer OS was seen with several patient subgroups; including patients with higher albumin, lower neutrophil, and lower LDH levels, those without a prior history of docetaxel, patients with an EOD of 1/2, and patients with higher hemoglobin, lower ALP and lower PSA levels; however, it is important to note that these findings are not conclusive because they were selected by arbitrary cutoff (hazard ratio or its reciprocal < 0.7). These trends were generally consistent with previously reported prognostic factors in patients with mCRPC [22–26]. A trend for longer OS with lower neutrophil counts in the present study is likely to reflect the positive prognostic effect of lower NLR (neutrophil–lymphocyte ratio), which has been reported in several malignancies including prostate cancer [27, 28]. The trend for longer OS with lower NLR may also be due to the increased immunity seen in patients lower NLR, which may in turn increase the efficacy of therapy by facilitating an abscopal effect, where a response is observed in lesions that are distant from the site of treatment. This effect has been seen with both radiotherapy [29, 30], and, more recently, studies have suggested it may occur with radium-223 [31, 32]. Regarding prior docetaxel, the trend for OS observed in this study is similar to ALSYMPCA, in which median OS with radium-223 was numerically longer in patients without prior docetaxel (with vs without prior docetaxel: 14.4 vs 16.1 months); however, the hazard ratio of radium-223 to placebo was similar (0.70 vs 0.69) regardless of prior docetaxel [33]. It can be concluded that, in the present study, OS was affected by generally similar factors to those seen in previous studies.

The present analysis had a number of limitations. Firstly, the study had a single treatment arm with a relatively small sample size designed for the evaluation of ALP change; therefore, it was underpowered for other outcomes, including safety, OS, and the subgroup analyses. Furthermore, follow-up was limited to 3 years, which may not be long enough to fully assess the number of treatment-related malignancies. Additional studies with larger sample size are needed to confirm the long-term safety. At present, an international, prospective, observational, single-arm study (Radium-223 alpha Emitter Agent in non-intervention Safety Study in mCRPC popUlation for long-teRm Evaluation, REASSURE; NCT02141438), in which patients will be followed until 7 years after the last radium-223 dose, is ongoing.

In conclusion, in this 3-year follow-up of Japanese patients with symptomatic mCRPC and bone metastases, no new safety concerns were identified and no second primary malignancies were reported, suggesting that radium-223 has a favorable long-term safety profile in Japanese patients. Median OS with radium-223 was at least as favorable as that observed in ALSYMPCA. When considered together with the decrease in ALP levels associated with radium-223 and reported in the primary analysis, it is likely that the efficacy findings of the present study are consistent with those of ALSYMPCA. The safety and efficacy results of the present study suggest that radium-223 provides long-term benefits in Japanese patients with mCRPC and bone metastases.

## Electronic supplementary material

Below is the link to the electronic supplementary material.

Supplementary material 1 (DOCX 41 KB)
