# Supplementary material for: Three-year follow-up of a phase II study of radium-223 dichloride in Japanese patients with symptomatic castration-resistant prostate cancer and bone metastases
Source: Int J Clin Oncol. 2019 Mar 14;24(5):557–66. doi: 10.1007/s10147-018-01389-4 (PMC6469691; doi:10.1007/s10147-018-01389-4)
Supplement: Supplementary file 1 — Supplementary material 1 (DOCX 41 KB) [file 10147_2018_1389_MOESM1_ESM.docx]

# Supplementary Materials

**Supplementary Table 1.** Anticancer drugs, which were started within 30 days after the last radium-223 injection.

| **Treatment** | **N** |
| --- | --- |
| Docetaxel | 1 |
| Enzalutamide | 12^a^ |
| Abiraterone (plus dexamethasone) | 1 |
| Bicalutamide | 1 |
| Prednisolone | 4^b^ |
| Dexamethasone (monotherapy) | 1 |
| Betamethasone | 1 |
| Strontium-89 | 1^c^ |

^a^Includes two cases started 33 and 35 days after the last radium-223 injection.

^b^Includes case started 66 days after the last radium-223 injection.

^c^Treated 42 days after the last radium-223 injection.

**Supplementary Table 2.** Adverse events not related to study drug and reported during follow-up.

| **Adverse event** | **N^a^** | | |
| --- | --- | --- | --- |
|  | **Grade1–2** | **Grade3** | **Serious** |
| Renal impairment |  | 1 | Yes |
| Hypophosphatemia, syncope |  | 1 each | No |
| ALT increased, AST increased, constipation, fracture, GGT increased, gum infection, tumor pain, weight loss | 1 each |  | No |

^a^Reporting of non-related AE was not mandated in protocol, but some were done voluntarily. Incidence was not calculated because the numbers are not comprehensive.

ALT, alanine aminotransferase; AST, aspartate aminotransferase; GGT, γ-glutamyl transpeptidase.

**Supplementary Table 3.** Characteristics of patients who experienced fracture with radium-223 dichloride treatment.

|  | **Patient 1** | **Patient 2** | **Patient 3** |
| --- | --- | --- | --- |
| Age, years | 81 | 67 | 74 |
| Preferred term | Spinal compression fracture | Spinal compression fracture | Pathological fracture |
| Reported term | Compressed fracture of eleventh dorsal vertebra | Collapsed vertebra | Pathological fracture (Sciatic, right iliac, lumbar spine L5 left transverse process) |
| Serious | No | No | No |
| CTCAE grade | Grade 2 | Grade 2 | Grade 2 |
| Number of radium-223 injections | 6 | 4 | 4 |
| Time from first injection, days | 6 | 47 | 131 |
| Time from last injection, days | Not applicable | Not applicable | 47 |
| Treatment emergent | Yes | Yes | No |
| Drug-related | No | No | No |
| Dose modification/interruption of radium-223 | None | None | Not applicable |
| Outcome | Not resolved | Not resolved | Not resolved |
| Prior chemotherapy | None | Yes | Yes |
| Prior/concomitant BMA | None | None | None |
| Prior corticosteroid | Yes | Yes | Yes |
| Prior new hormonal agents | Abiraterone^a^ | None | None |
| New hormonal agents after radium-223 and prior to fracture | Not applicable | Not applicable | Enzalutamide^b^ |
| Prior history of fracture | None | None | None |

^a^Continued for 111 days and ended 361 days before first injection of radium-223

^b^Started 18 days after the last injection of radium-223 , continued for 29 days, and ended on day before the fracture

BMA, bone-modifying agents; CTCAE, Common Terminology Criteria for Adverse Events.
